# Assessing the Generalizability of Client Experience Measurement Tools in Low- and Middle-Income Countries: A Narrative Review

**DOI:** 10.9745/GHSP-D-23-00364

**Published:** 2025-12-31

**Authors:** Andrew Corley, Susannah Gibbs, Nirali Chakraborty, Lara Fields, Giannina Chávez Ackermann, Jasmine Coulson, Yixin Zhang, Paul Bouanchaud

**Affiliations:** a Metrics for Management, Baltimore, MD, USA.; b Population Services International, Washington, DC, USA.; c Metrics for Management, Baltimore, MD, USA. Now with Art & Science Group, Baltimore, MD, USA.; d London School of Economics, London, England.

## Abstract

**Introduction:**

The experiences of people who interact with a health system form a key component of overall quality of care in that system. Yet, client experience is rarely reflected in how health systems are designed and assessed. To make meaningful progress on delivering high-quality patient-centered care, health systems actors need valid measures of client experience of care. However, no cross-cutting measure of client experience of care exists at present that could facilitate measurement and benchmarking across multiple health service areas.

**Methods:**

We conducted a phased literature search using multiple scholarly databases to identify peer-reviewed articles detailing the development, validation, or adaptation of measures relating to the concept of client experience in sexual and reproductive health care, HIV, primary care, noncommunicable disease management, and health services management and marketing. Measure domains were thematically analyzed and mapped against domains of an existing client experience of care framework—effective communication, respect and dignity, and emotional support.

**Results:**

We identified 73 articles that met inclusion criteria and that recounted the development, validation, or adaptation of 61 different measures of health care quality and responsiveness. Numerous measures exhibited significant overlap with an existing conceptual framework for client experience, but few measures were used across health areas.

**Discussion:**

Content of many of the measures identified in this review mapped closely to domains that appear in an existing framework for client experience of care, including effective communication, respect and dignity, and emotional support. These findings support the notion that developing a generalizable measure of client experience of care could be technically feasible.

## Abstract (precis)

Existing measures of person-centered health care quality and responsiveness, across multiple health areas, share many conceptual similarities with the concept of client experience of care, suggesting that developing a cross-cutting, common measure of client experience is possible.

## Teaser

**Key Findings:**

- Numerous measures of client experience of care have sought to assess the quality of health services from clients’ perspective, frequently using related, overlapping theoretical domains.
- No measures identified through this review possessed the attributes necessary to be adopted for use as a generalizable measure of client experience of care across multiple health areas.
- The overlapping nature of many of the measures’ constituent domains across different health areas suggests that it would be feasible to develop a cross-cutting measure of client experience of care.

**Key Implication:**

- There is an opportunity to develop a new measure of client experience of care that would permit health system actors, including policy officials, program managers, and funders, to compare service quality across health areas and points of service delivery.

## INTRODUCTION

The Sustainable Development Goals (SDGs) prioritize the development of systems of universal coverage of high-quality essential health services.^1^^,^^2^ This is especially relevant to low- and middle-income countries (LMICs) in which greater emphasis has been placed on the quality of services and patient-centeredness of these services.^2^^–^^4^ People’s care experience has become widely recognized as a foundational element to the provision of high-quality health services for the value it places on delivering humane, respectful care and for its direct and indirect effects on clinical effectiveness and patient safety.^5^^–^^8^ Despite its importance, client experience is rarely reflected in how health systems are designed and assessed. To make meaningful progress on delivering high-quality patient-centered care, health systems actors need valid measures of client experience of care.

In this article, we choose to employ the term “client” instead of “patient” when discussing experience of care. This choice reflects the term’s wider suitability across different states of health, person-provider relationships, and health delivery channels. While various organizations and researchers have conceptualized client experience of care in different ways,^9^^–^^11^ the common thread woven through all these definitions is that client experience encompasses the spectrum of interactions that a person may have with a health care system across the continuum of care that influence their perceptions of the quality of that care.

**FIGURE 1:**
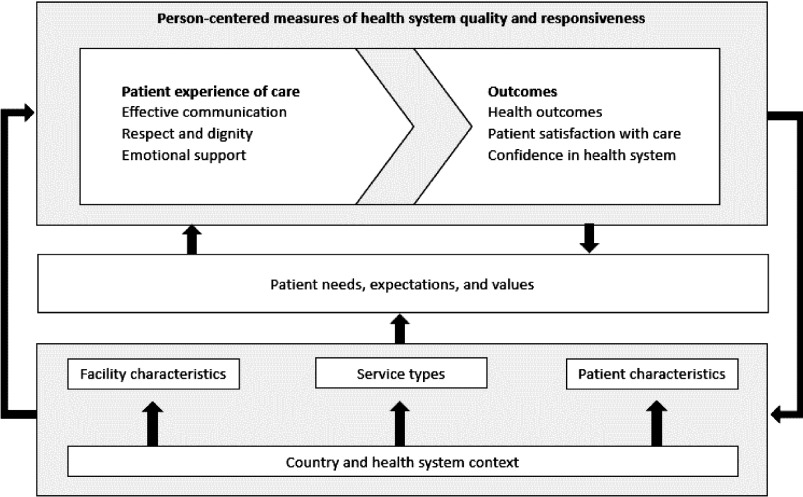
Conceptual Framework for Person-Centered Measures of Health System Quality and Responsiveness^a^ ^a^ Source: Larson et al., 2019^12^

A widely accepted conceptual model for client experience of care has yet to be developed, leaving open the need for further research into the constituent dimensions and interactions that shape an individual’s perception of their care journey. However, Larson and colleagues have proposed that client experience of care is broadly composed of 3 domains: effective communication; respect and dignity; and emotional support (Figure 1).^12^ Similar domains appear in related frameworks for health care quality, including the World Health Organization’s maternal quality of care framework^13^ and Judith Bruce’s family planning quality framework.^14^ Within the Larson model, patient needs, expectations, and values, along with interpersonal and facility-level factors such as the ease of seeking care or obtaining appointments, the availability of pertinent information, and the quality of communication with health care providers and administrative staff can all affect a client’s experience of care. Although frameworks such as the World Health Organization’s maternal quality of care framework and Judith Bruce’s family planning quality framework are useful for specific health areas, Larson and colleagues’ framework was selected for this literature review because it provides a more generalized primer for understanding the constituent elements of client experience of care, offering a helpful foundation for the development of a more refined measurement approach.

Considered from a rights-based perspective alone, all people deserve to receive care characterized by autonomy, dignity, respect, and emotional support.^3^ However, the concept of client experience takes on even greater saliency for its association with improved health outcomes;^15^^–^^17^ greater satisfaction and confidence in one’s health system;^3^^,^^18^ and improved clinical effectiveness and increased patient safety.^6^ Despite the importance of patient-reported measures of health care quality and their relevance to essentially all areas of health service delivery, to our best knowledge there exists no common or standard approach to measuring client experience of care that is widely used in multiple health areas across LMICs. This fragmented approach to measuring client experience fails to capture the fundamental reality of how patients actually experience health care.^3^

From a client’s perspective, a health facility is a holistic environment where they seek care, not a collection of disconnected service areas. When clients visit a health post, they are not thinking about separate domains or categorical metrics; they are experiencing an integrated journey of receiving care. A client-centered approach to measuring service quality recognizes this holistic experience. While different health areas possess unique characteristics that may influence clients’ experiences, there are common features of client experience that transcend these domain-specific distinctions. Leveraging these commonalities and facilitating knowledge and best-practice sharing across health areas is more likely to occur when a common measurement approach is available.

Action-oriented measurement is central to learning health systems.^3^ A generalizable approach to measuring client experience of care could offer substantial advantages to health systems. First, it would provide standardized and comparable assessment tools that could transcend specific health area, geographic, and cultural boundaries. By utilizing a set of common measures, one could obtain consistent data across an array of health areas and geographies, enabling meaningful comparisons and identification of patterns and trends. Doing so would facilitate the identification of best practices and areas for improvement. This evidence could similarly inform the development and implementation of more effective and contextually appropriate quality improvement interventions. Furthermore, a generalizable approach to measuring client experience of care would facilitate accountability and transparency.

This review aims to identify measures and domains that possess broad geographic and health area relevance, thereby enhancing our understanding of the essential features necessary for a comprehensive, broadly applicable approach to measuring client experience of care. By analyzing existing measures related to client experience of care, we can identify common themes and domains that resonate across diverse populations and health care settings. The objectives of this narrative review are to examine the domains encompassed in existing measures of health care service experience, person centeredness, and satisfaction with care used across 6 areas of health services and to describe how these measures have been tested and used across multiple health areas and geographic contexts. This process will inform the development of a new measure that encompasses crucial aspects of client experience while remaining adaptable to various contexts.

## METHODS

A narrative review methodology was employed to synthesize and summarize evidence on existing measures related to client experience among adult and pediatric client populations in 6 health areas: malaria, sexual and reproductive health (SRH), HIV, primary care, noncommunicable diseases (NCDs), and health services marketing and management. No limitations were placed on the type of facility in which measures were intended for use. Primary care refers to models of health care that facilitate accessible first-contact care designed to optimize population health.^19^ A narrative review approach to our objectives was chosen because the method allows authors a means to conduct a scholarly summary, interpretation, and critique of the available literature with the overall goal of crafting an authoritative and convincing argument.^20^

Given the heterogeneity of topic areas, a phased approach was taken to our database searches. An initial rapid review was conducted in 2021 on malaria, SRH, HIV, and primary care health areas.^21^ Databases searched during this phase included PubMed, Web of Science, and Global Index Medicus. An expanded follow-on review of the same health areas—malaria, SRH, HIV, and primary care—was conducted in 2023 in the same databases as well as Ovid MEDLINE. Literature exploring the development and validation of person-centered measures in NCD care was conducted in 2023 in PubMed and Web of Science. Lastly, owing to their business and marketing focus, ABI/INFORM and Business Source Ultimate, 2 of the most comprehensive databases on marketing and management research, were searched in 2023 to identify literature related to measures developed for use in health services marketing and management.

Our review considered peer-reviewed studies published between January 2000 and January 2023. We considered quantitative, qualitative, and mixed-methods research focused on client experiences in malaria, SRH, HIV, primary care, and NCDs. We also included studies exploring client experience for the purposes of improving health services marketing and management. Studies were eligible if they reported on the validation of a new client experience measure or the adaptation and validation of an existing measure in a novel population or cultural context in adult patient populations. Measures were included if they were interpreted as reflecting client experiences by the publications’ authors. Additionally, to supplement our initial literature search, we employed a snowball approach by examining the reference lists of the identified articles to identify additional relevant literature.

The client experience search terms used included ‘experience of care,’ ‘care experience,’ ‘patient experience,’ ‘user experience,’ ‘client experience, and ‘consumer experience.’ In the second review conducted into the malaria, SRH, HIV, and primary care health areas, the term ‘patient centered care’ was also included. To these were added search terms specific to each health area. Given the depth of literature known to emanate from LMICs in the health areas of malaria, SRH, HIV, and primary care, an LMIC filter was added to these searches. This same filter was not applied to searches for measures related to NCD care and health services marketing and management because of concerns that much of the research in these 2 areas continues to be conducted primarily in upper-income countries. Search terms were adapted as appropriate to the 5 databases.

One reviewer screened the titles and abstracts of identified articles to determine their relevance. Full-text articles meeting the inclusion criteria were retrieved and assessed for eligibility. The reviewers critically appraised the selected articles to evaluate their relevance and contribution to the topic. Data extraction was conducted using standardized data capture forms designed to collect relevant information from the selected articles. This included study characteristics (e.g., study design, sample size, setting), measure attributes, and domains. The extracted data were analyzed thematically and synthesized to identify gaps in the literature.

To compare measures’ overlapping and complementary domains to those theorized to make up the construct of client experience of care, existing measures’ domains were mapped to 3 client experience of care sub-domains—effective communication, respect and dignity, and emotional support—as defined in Larson and colleagues’ “Framework for person-centered measures of health system quality and responsiveness.”^12^ This exercise allowed the authors to examine how existing measures of client experience relate to and contrast with a prevailing conceptualization of client experience of care and to observe how measures of these domains have been adopted for use across health areas.

## RESULTS

In this narrative review, we identified a total of 73 articles that met our inclusion criteria. These articles collectively covered 61 different measures of client experience. Table 1 describes the number of measures by health area as well as the number of citations describing these measures. Table 2 summarizes the domains extracted from the measures and illustrates how, among those that were found to be conceptually similar to Larson and colleagues’ (2018) client experience of care domains, these domains were categorized. Table 3 provides the measure names; the countries in which available literature describes their development, validation, or adaptation; and how each measure’s domains overlap with those of our conceptual framework for client experience of care’s domains. Measures in Table 3 are classified by the health area search in which they were identified.

**TABLE 1.** Number of Client Experience Measures and Citations in Included Articles, by Health Area

**Health Area**	**No. of Measures**	**No. of Citations**
Sexual and Reproductive Health	12	14
HIV	3	4
Primary Care	2	6
Noncommunicable Diseases	21	26
LMICs	2	4
HICs	19	22
Health Services Marketing and Management	23	23
LMICs	6	6
HICs	17	17
**Total**	**61**	**73**

Abbreviations: HICs, high-income countries; LMICs, low- and middle-income countries.

**TABLE 2.** Client Experience Domains of the Measurement Tools in the Included Articles and Their Overlap With Larson and Colleagues’ Domains^a^

**Client Experience of Care Domains**		
**Effective Communication**	**Respect and Dignity**	**Emotional Support**
Access to Information Care Teams Across Settings Clarity of Information Communication Communication and Autonomy Communication with Nurses and Doctors Continuity of Care Coordinated and Comprehensive Care Coordination Coordination of Care Decision Support Diagnosis Education and Shared Knowledge Effective Use of Method Eliciting Client’s Preferences Financial Advice Follow-up/Coordination Free Flow and Accessibility of Information General Practitioner Involvement Goal Setting/Tailoring Health Information and Decision-Making Support Information and Questions Information Exchange Information for Treatment Decision-Making Information of Care Pathway Information on Changes Related to Illness Information Services Managing Appointments Method Selection Patient Activation Person-Focused Care Over Time Problem Solving Providing General Information Providing Specific Information Provision of Information Rapport Receiving Adequate Information Suspicion of Diagnosis Symptom Non-reporting	Abuse Abuse-Free Care Accessibility of Care Accessing Support Attitude and Commitment of Service Providers Autonomy Care Goals for Patients Conduct of Healthcare Professionals Confidentiality Cultural Competence Decision-Making About Treatment Dignity Discrimination Discriminatory Behavior Friendliness Interpersonal Connection Interpersonal Relationship Making Treatment Decisions Non-Discrimination Patient-Centered Approach by Doctors Patient-Centeredness Physical Abuse Privacy Quality of Life Respect Respectful and Engaging Interaction Respectful and Supportive Care Respectful Care Respectful Coordinated Care Stigma Stigma and Discrimination Verbal Abuse	Activities to Address Biopsychosocial Needs Comfort Disclosure Support Family-Centeredness Feelings of Abandonment Provide Social Support Psychosocial Care and Aftercare Psychosocial Needs Sharing Feelings with Others Social Support Supportive Care Sustaining Normality Trustful Relationship with Health Care Staff Value for Non-Provider Social Support Worries and Anxieties

a Larson and colleagues^12^ proposed that client experience of care is broadly composed of 3 domains: effective communication, respect and dignity, and emotional support.

**TABLE 3.** Client Experience Measures in Included Articles, by Health Area and Measure Domains

**Measure Name or Study Description**	**Countries Validated**	**Population(s)**	**Client Experience of Care Domains**			**Other Domains Addressed**	**References**
			**Effective Communication**	**Respect and Dignity**	**Emotional Support**		
**Sexual and Reproductive Health**							
Person-Centered Maternity Care (PCMC)	India Kenya	Women who recently gave birth in a health facility	Communication and Autonomy	Dignity and Respect	Supportive Care		^22^ ^,^ ^23^
PCMC short	Kenya Ghana India	Women who recently gave birth in a health facility	Communication and Autonomy	Dignity and Respect	Supportive Care		^24^
Bohren et al. (2018)	Nigeria Ghana Guinea Myanmar	Women who gave birth in the past 8 weeks	Communication	Physical Abuse; Verbal Abuse; Stigma; Discrimination	Supportive Care	Failure to Meet Professional Standards; Neglect and Abandonment; Pain Relief	^25^ ^,^ ^26^
Gurung et al. (2021)	Nepal	Women giving birth at a public hospital providing comprehensive emergency obstetric and neonatal care	Rapport	Abuse; Stigma and Discrimination		Standard of Care; Care Not Refused Due To Finances	^27^
QCC (Quality Contraceptive Counselling) Scale	Mexico	Health facility clients interested in learning about contraception during their visit	Information Exchange	Disrespect and Abuse; Interpersonal Relationship			^28^
Jain et al. (2019)	India	Married women adopting a long-acting reversible contraceptive method	Method Selection	Respectful Care		Continuity of Contraceptive Care Use Effective Use of Method	^29^
IQFP (Interpersonal Quality of Family Planning) scale	India	Young married couples	Receiving Adequate Information	Interpersonal Connection	Decision Support		^30^
Net Promoter Score (NPS)	India Kenya Nigeria El Salvador	Family planning clinic clients				General (e.g., likelihood of recommending this clinic to someone)	^31^
Respectful Maternity Care (RMC) Scale	Ethiopia	Women who gave birth in the past 7 weeks		Non-Discrimination; Abuse-Free Care; Friendliness		Timeliness of Care	^32^
Person-Centered Family Planning (PCFP) Scale	India Kenya	Women seeking family planning services at public health facilities	Communication	Autonomy; Respectful Care		Health Facility Environment	^33^
Person-Centered Abortion Care (PCAC) Scale	Kenya	Women who received an abortion-related service	Communication and Autonomy	Respectful and Supportive Care			^34^
Quality of Family Planning Counselling (QFPC) Measure	India	Family planning clients	Provision of Information; Eliciting Client’s Preferences	Respectful and Engaging Interaction			^35^
**HIV**							
Health System Responsiveness Survey	Tanzania	Adults living with HIV currently on antiretroviral therapy	Communication	Respect; Confidentiality	Comfort	Access; Perceived Quality	^36^
CARE’s Community Score Card (CSC)	Malawi	Pregnant and lactating people living with HIV		Attitude and Commitment of Service Providers; Discriminatory Behavior; Confidentiality	Disclosure Support		^37^
Quality of Care Through the Patient's Eyes - HIV (QUOTE-HIV)	Brazil The Netherlands	Clients living with HIV receiving outpatient care	Communication; Access to Information	Respect; Dignity; Privacy; Autonomy	Social Support	Facilities; Time	^38^ ^,^ ^39^
**Primary Care**							
Primary Care Assessment Tool (PCAT)	United States Canada Brazil Spain South Korea China Taiwan Tibet Vietnam South Africa Malawi	Primary care clients	Person-Focused Care Over Time; Coordination	Cultural Competence	Family-Centeredness	First Contact Care; Comprehensiveness; Community Orientation	^40^ ^–^ ^45^
Patient Assessment of Healthcare for Outpatient Care (O-PAHC)	Ethiopia	Adults receiving outpatient care at hospitals or health centers	Communication with Nurses and Doctors			Physical Environment	^46^
**Noncommunicable Diseases**							
Chronic Cancer Experiences Questionnaire (CCEQ)	United Kingdom	Patients with breast, gynecological, colorectal, renal, or prostate cancer	Information and Questions; General Practitioner Involvement; Financial Advice; Managing Appointments; Coordination of Care; Symptom Non-reporting	Making Treatment Decisions; Accessing Support	Sharing Feelings with Others; Worries and Anxieties; Sustaining Normality	Clinical Trials	^47^
Consumer Quality Index Breast Cancer (CQI-BC)	The Netherlands	Patients with breast, lung, colorectal, prostate, hematological, gynecological, or skin cancer	Information Services; Continuity of Care	Conduct of Healthcare Professionals; Accessibility of Care; Autonomy	Psychosocial Care and Aftercare	Expertise of Healthcare Professionals; Hospital Facilities; Time Schedule	^48^
Consumer Quality Index Cancer Care (CQI-CC)	The Netherlands	Patients with breast, lung, colorectal, prostate, hematological, gynecological, or skin cancer	Education and Shared Knowledge; Free Flow and Accessibility of Information	Patient-Centered Approach by Doctors		Skills & Cooperation of Healthcare Professionals; Collaboration & Team Management	^49^
LifeCourse Experience Tool	United States	Patients with heart failure, cancer, or dementia	Care Teams Across Settings; Communication	Care Goals for Patients			^50^
Measure of Processes of Care for Adults (MPOC-A)	Canada	Patients with joint or hip replacements	Providing General Information; Providing Specific Information; Coordinated and Comprehensive Care	Respectful and Supportive Care			^51^
Opportunity for Treatment In Oncology (OPTION) Questionnaire	Italy	Patients with breast or colorectal cancer	Information of Care Pathway; Information on Changes Related to Illness		Feelings of Abandonment; Trustful Relationship with Health Care Staff	Collaboration Among Health Care Professionals	^52^
Pulmonary Arterial Hypertension Clinic - Patient Reported Experience Measurement (PAH-PREM)	Sweden	Patients with pulmonary arterial hypertension	Communication	Patient-Centeredness		Effectiveness; Timeliness	^53^
Patient Assessment of Cancer Communication Experiences (PACE)	Portugal	Oncology patients		Decision-Making About Treatment		Surgery; Chemotherapy; Radiation Therapy; Suspicion of Diagnosis; Diagnosis	^54^
Patient Assessment of Chronic Illness Care (PACIC)	Denmark The Netherlands United States France	Patients with cardiovascular disease or diabetes mellitus	Patient Activation; Goal Setting/Tailoring; Problem Solving; Follow-up/Coordination			Delivery-System/Practice Design	^55^ ^–^ ^58^
Short version of the Patient Assessment of Chronic Illness Care (PACIC-M11)	Malaysia	People with type 2 diabetes or hypertension in primary care settings	Patient Activation; Goal Getting/Tailoring			Delivery System Design/Practice Design	^59^ ^–^ ^61^
Older Patient Assessment of Chronic Illness Care (O-PACIC) Sale	The Netherlands	Recently discharged hospitalized patients	Patient Activation; Goal Setting/Tailoring; Problem Solving/Contextual; Follow-Up Coordination			Delivery-System/Practice Design	^62^
Patient Satisfaction with Cancer-Related Care (PSCC)	United States	Patients with breast, cervical, colorectal, or prostate cancer	Communicational/ Informational; Coordination of Care	Interpersonal/ Relational		Access/Logistical	^63^
Patient-Centered Quality of Cancer Care Questionnaire (PCQCCQ-S)	Mexico	Oncology patients	Clarity of Information; Information for Treatment Decision-Making	Respectful Coordinated Care	Activities to Address Biopsychosocial Needs	Timely Care	^64^
Patients and the Cancer Care Experience (PCCE)	United States	Oncology patients	Health Information and Decision-Making Support	Quality of Life	Provide Social Support; Psychosocial Needs; Value for Non-Provider Social Support		^65^
Quality of Care Through the Patient's Eyes (QUOTE)	The Netherlands	Oncology patients	Treatment-related Information; Prognosis Information; Rehabilitation Information; Interpersonal communication; Tailored Communication; Affective Communication		Coping Information		^66^
Quality of Patient-Centered Cancer Care (QPCCC)	Australia	Hematology cancer patients	Provision of Information, Communication and Education; Coordinated and Integrated Care	Patient Centeredness; Safety; Equity	Emotional Support; Involvement of Family and Friends	Physical Comfort; Effectiveness; Timeliness; Efficiency	^67^
CONTACT-Patient-Centered Care Questionnaire (CONACT-PCCQ)	Belgium	Oncology patients	Information, Communication and Education; Coordination of Care	Respect for the Patient’s Values, Preferences and Expressed Needs	Emotional Support; Involvement of Family and Friends	Physical Comfort	^68^
Patient Experience Survey (PES)	Canada	Radiation therapy patients	Appointment Scheduling	Interprofessional Staff/Patient Encounters		Same Day Waits; Hospital/Waiting Room Environment; Patient Care; Weekly Oncologist Review; Parking	^69^
Patient Centered Communication in Cancer Care (PCCCC)	United States	Patients with colon or rectal cancer	Exchanging Information; Fostering Health Relationships; Making Decisions; Managing Uncertainty	Enabling Patient Self-Management	Responding to Emotions	Cross-Cutting Items	^70^
Patient-Centered Measures of End-of-Life Care Quality for Children with Cancer	United States	Pediatric oncology and palliative care patients	Communication	Meeting Patient Preferences; Symptom Management		Healthcare Use; Interdisciplinary Care	^71^
Patient-Centered Primary Care	The Netherlands	Patients with multiple chronic conditions	Information and Education; Continuity and Secure Transition between Healthcare Settings; Coordination of Care	Respect for Patients’ Preferences	Emotional Support; Involvement of Family and Friends	Access to Care; Physical Comfort	^72^
**Health Services Marketing & Management**							
Health Service Quality Scale	Australia	Outpatient oncology clinic and primary care clinic clients	Interpersonal Quality			Technical Quality; Environment Quality; Administrative Quality	^73^
Emergency Room Service Quality	Israel	Individuals accompanying emergency department patients			Staff Caring	Staff Professionalism; Tangibles	^74^
Health Service Quality Scale	Colombia	Outpatient health clinic clients	Patient-Centered Communication			Process Quality	^75^
Continuity Quality of Care Indicator	Poland	Outpatient health clinic clients	Informational Continuity; Cross-Boundary and Team Continuity	Patient Empowerment	Relational Continuity	Managerial Continuity; Flexible Continuity; Longitudinal Continuity	^76^
Alberta Continuity of Services Scale-Mental Health (ACSS-MH)	Canada	In- and outpatient mental health service clients		Individualized Care		Responsive Caregiver Responsive System	^77^
The Humanistic Relationship Importance Scale	Canada	Chronic care facility patients		Recognizing and Supporting Choice; Supporting Human Uniqueness	Relational Availability; Forming Connections	Promoting Quality of Daily Life	^78^
Parent Satisfaction Scale (PSS)	United States	Pediatric mental health treatment clients		Met Expectations		Met Desires; Met Needs	^79^
Responsiveness of Physician (ROP) Scale	Bangladesh	Rural health service clients	Informing and Guiding	Respecting	Friendliness	Financial Sensitivity; Gaining Trust	^80^
mHealth Service Quality Scale	Bangladesh	mHealth consumers	Information Quality		Interaction Quality	System Quality	^81^
Multidimensional Scale for Healthcare Service Quality (HCSQ)	India	Medicine, surgery, pediatric, and gynecology inpatients	Interaction Quality			Physical Environment Quality; Outcome Quality	^82^
Evaluation of Client Services (ECS)	United States	Outpatient mental health treatment services clients	Communication and Information Exchange		Treatment Relationship	Treatment Management and Outcome; Reachability of Treatment Facilities	^83^
The Health Service Quality (HEALTHQUAL) Measure	South Korea	Hospital in- and outpatients			Empathy	Tangible; Safety; Efficiency; Care Service Improvements	^84^
Medical Tourism Experience (MTEX) Scale	India	Medical tourism clients		Medical Service Quality		Treatment Quality; Medical Tourism Expenses; Medical Tourism Infrastructure; Destination Appeal; Destination Culture; Ease of Access	^85^
Cultural Differences in Healthcare	South Korea	Medical tourism clients	Communication	Cultural Values; Religion		Hospital Care and Services; Food; Healthcare System; Facility	^86^
Scale for e-Health Service Quality	Switzerland	mHealth consumers	Information		Empathy; Individualization; Ethical Conduct	Accessibility; Competence; Usability; Security; System Integration; Trust; Performance; Reliability; Ability to Respond	^87^
Navigation Satisfaction Tool (NAVSAT)	Canada	Parent and guardians of youth receiving mental health and addiction services	Ability to Listen; Communication Frequency; Frequency of Contact	Confidentiality		Likelihood of Recommending Service; Overall Satisfaction; Navigator Helpfulness; Ability to Understand Mental Health System; Intake Procedures; Treatment Options Information; Appropriate Treatment Found; Impact on Family	^88^
The Acute Care Hospital Foodservice Patient Satisfaction Questionnaire (ACHFPSQ)	Australia	Acute care inpatients				Food Quality; Meal Service Quality; Staff Service Issues; Physical Environment	^89^
The Birth Satisfaction Scale (BSS)	United Kingdom	Postpartum women		Quality of Care Provision	Quality of Care Provision	Personal Attributes; Stress Experienced During Labor	^90^
The Cataract Service Satisfaction Tool	United Kingdom	Outpatient cataract surgery clients	Collaboration With Doctors and Nurses; Quantity and Quality of Information	Autonomy; Empathy		Knowledge; Facilities; Waiting Times; Overall Satisfaction; Ability to Manage at Home; Access to Postoperative Support	^91^
Clinical Decision-making Involvement and Satisfaction (CDIS) Scale	Germany; England; Italy; Hungary; Switzerland	Community-based mental health service clients		Involvement		Satisfaction	^92^
Key Quality Characteristics Assessment for Hospital (KQCAH) Scale	United States	Recently discharged hospitalized patients	Information	Respect & Caring		Effectiveness & Continuity; Appropriateness; Efficiency; Effectiveness-Meals; First Impression; Staff Diversity	^93^
Chinese Patients' Satisfaction Scale (C-PSS)	Taiwan	Hospital outpatient clients		Respect	Warm Interactions	Efficiency; Fairness; Professionalism; Responsibility	^94^
Responsiveness of Physicians Scale (ROP-Scale)	Bangladesh	COVID hospitalized patients	Informativeness	Courteousness		Trustworthiness	^95^

### Sexual and Reproductive Health

The review identified 12 measures developed, validated, or adapted to measure the person-centeredness of many SRH services in various country contexts. Measures assessing the person-centeredness of maternal care services were identified with the greatest frequency,^22^^–^^24^^,^^26^^,^^27^^,^^32^ followed by those assessing contraceptive care quality.^28^^–^^31^^,^^33^^,^^35^ One measure included in the review was designed to evaluate the person-centeredness of abortion care services.^34^ Many of the domains of these measures mapped onto the client experience of care framework. In all but 2 cases, measures contained domains aligning with effective communication. All but one measure included domains that aligned closely with respect and dignity. Despite these areas of overlap, only 4 measures contained domains related to emotional support. Three measures, including domains related to the concept of emotional support, were designed to evaluate the quality of maternal care while the remaining measures were devoted to assessing contraceptive care quality.^22^^–^^26^^,^^30^

In regard to domains not classifiable within the 3 domains of our conceptual framework for client experience of care, 2 measures included domains devoted to assessing whether providers met professional standards or recognized standards of care.^25^^–^^27^ Other domains that did not map directly onto the client experience of care included domains devoted to neglect, pain management,^25^^,^^26^ continuity of care,^29^ affordability,^27^ and overall satisfaction,^31^ timeliness of care,^32^ and health facility environment.^33^

### HIV

We identified 3 measures in development, validation, or adaptation studies for use in HIV/AIDS care. A 2014 study reported on the development of a health system responsiveness survey in Tanzania for use with adults living with HIV who were on antiretroviral therapy.^36^ The survey includes domains that address effective communication, respect and dignity, and emotional support. Other domains included access and perceived quality.

The second measure was developed using CARE’s Community Score Card (CSC), a widely used approach for participatory community assessment and empowerment, with pregnant and breastfeeding women living with HIV in Malawi.^37^^,^^96^ Domains included in this measure align with the client experience of care framework’s domains of respect and dignity and emotional support.^97^ Finally, QUOTE-HIV,^38^^,^^39^ a measure of care quality reported from a patient’s perspective, contains measurement domains aligning with the client experience of care domains of effective communication, respect and dignity, and emotional support. The QUOTE-HIV also includes domains related to facility quality and waiting times.

### Primary Care

We identified validation studies for 2 measures in primary care. Originally developed in the United States,^98^^,^^99^ the Primary Care Assessment Tool (PCAT) has since been adopted in at least 10 other countries, including Brazil, China, Malawi, South Africa, South Korea, Spain, Taiwan, Tibet, and Vietnam.^41^^–^^45^^,^^100^ The PCAT is designed for use in primary care settings, particularly community health centers. Its domains overlap with the client experience of care framework domains, and it also includes domains related to first contact care, comprehensiveness, and community orientation. The Patient Assessment of Healthcare for Outpatient Care (O-PAHC), which was adapted for use in Ethiopia among adults receiving outpatient care at hospitals or health centers, contains domains that map onto the effective communication domain as well as additional domains of quality that evaluate facilities’ physical environments.^46^

### Noncommunicable Diseases

We identified 21 patient-reported measures of service quality for use in NCD care and management. Only 2 of the 20 instruments were validated in patient populations in LMICs;^61^^,^^64^ the remainder were validated for use in European or North American countries. The specific NCD area of most intense inquiry was cancer, with two-thirds of referenced studies devoted to measuring care quality for breast, colorectal, blood, prostate, lung, and skin cancers.^47^^–^^50^^,^^52^^,^^54^^,^^63^^–^^71^ However, we also identified measures devoted to patients managing other chronic conditions such as type 2 diabetes mellitus, cardiovascular disease, pulmonary disease, dementia, and orthopedic conditions.^51^^,^^55^^–^^58^^,^^61^^,^^72^

The majority of measures were developed and validated within a single setting and against localized treatment populations. Only one tool, the Patient Assessment of Chronic Illness Care (PACIC) questionnaire, was validated and adapted to measure outpatient chronic care experiences in more than one country context. PACIC was developed to measure quality of care for in patients with type 2 diabetes or cardiovascular disease and was tested in Danish, Dutch, American, and French patient populations.^55^^–^^58^^,^^62^ The measure has also been adapted for use among Malaysian patient populations.^59^^–^^61^ Measurement domains of the PACIC overlap with the client experience of care framework domain of effective communication. The measures also include domains devoted to the design of delivery systems or practices.

Despite the variability of instruments present across the literature, the Institute of Medicine (IOM) Patient-Centeredness framework influenced a high proportion of the measures identified. Six measures—Quality of Patient-Centered Cancer Care (QPCCC); CONTACT-Patient-Centered Care Questionnaire (CONTACT-PCCQ); Patient Centered Communication in Cancer Care (PCCCC); Patient-Centered Measures of End-of-Life Care Quality for Children with Cancer; and Patient-Centered Primary Care—directly applied the IOM’s Patient-Centeredness framework to their design, leading to domains that showed a high degree of alignment across the domains of effective communication, respect and dignity, and emotional support.^64^^,^^67^^,^^68^^,^^70^^–^^72^ Other domains that appeared frequently in the instruments included timeliness of care, skills of medical professionals overseeing care, and the effectiveness of treatment.^48^^,^^49^^,^^53^^,^^69^

### Health Services Marketing and Management

We identified 23 articles describing the development and validation of 23 measures devoted to measuring both clinical and non-clinical components of health services marketing and management. Measures emanated from countries of varying levels of economic development. Six measures were developed in LMICs,^75^^,^^80^^–^^82^^,^^85^^,^^95^ while the remaining 17 were developed and validated in high-income countries.^73^^,^^74^^,^^76^^–^^79^^,^^83^^,^^84^^,^^86^^–^^94^ Measures devoted to evaluating the quality of acute inpatient and outpatient health services made up a majority of the articles identified.^73^^–^^80^^,^^82^^–^^86^^,^^88^^–^^92^^,^^94^^,^^95^ Among these, a handful of articles reported on the validation of measures meant to assess the quality of medical services from the perspective of foreign patients for purposes of evaluating medical tourism services,^85^^,^^86^^,^^94^ and one sought to assess specifically inpatient satisfaction with food service.^89^ Lastly, 2 measures of the informational quality and responsiveness of mHealth resources were also found.^81^^,^^87^

Most of the measures described in the articles overlapped conceptually with the client experience of care conceptual model. Thirteen measures reported measuring attributes of effective communication; 17 included concepts related to respect and dignity; and 8 considered emotional support in their service quality frameworks. One article describing the development of a scale designed to measure the quality of continuity of care among ambulatory patients in Poland had constituent domains that overlapped with all the domains of the client experience of care framework. Additionally, a number of measures included domains related to the cleanliness of the physical care environment.^73^^,^^74^^,^^82^^,^^84^^,^^91^ Along with domains aligning with the core experience of care domains, the measures also included domains devoted to technical and outcome quality.^73^^,^^75^^,^^79^^,^^83^^,^^87^ The inclusion of these domains support the observation that such measures of client satisfaction are frequently concerned with the end product of the client care journey.

## DISCUSSION

In our review of existing measures, we observed significant conceptual overlap with our framework for client experience. Nearly all the examined measures demonstrated partial, if not complete, alignment with our established domains of effective communication, respect and dignity, and emotional support. Beyond these core domains, our analysis revealed several additional key dimensions that warrant serious consideration in understanding clients’ experiences of health care services. The most prominently recurring dimensions across all health areas included facilities^33^^,^^46^^,^^69^^,^^82^^,^^86^^,^^91^ and care access,^36^^,^^63^^,^^72^^,^^83^^,^^85^^,^^87^ timeliness,^32^^,^^38^^,^^39^^,^^53^^,^^67^^,^^69^^,^^91^^,^^101^ and effectiveness.^29^^,^^53^^,^^67^^,^^82^^,^^93^

These dimensions consistently emerged as significant factors influencing client experiences, suggesting they are fundamental components of health service quality. This finding indicates that well-established dimensions from existing health service quality frameworks are equally vital when assessing health care services from a client-centered perspective.^13^^,^^102^^,^^103^ The recurring nature of these dimensions suggests they are not peripheral considerations but core elements that substantially contribute to clients’ overall experience and perception of health care quality.

There is growing recognition of the utility and need for generalizable measures of person-centered health service quality and responsiveness.^104^^–^^106^ As opposed to the current fragmented state of health area-specific measures, a generalized measurement approach that establishes a common framework and language can facilitate evaluation and discussion of health service quality across different programs. Widespread use of such measures to improve service delivery has the potential to contribute to the construction of more trustworthy, transparent, and responsive health systems.

While some measures exhibit significant overlap with our conceptual understanding of client experience, their development, validation, and use have been predominantly limited to specific health areas. As a result, very few measures have achieved widespread adoption across multiple health areas, and no single validated measure stands out as being well-suited of serving as a general, cross-cutting assessment of client experience in LMICs.

The limited generalizability of existing measures poses a challenge for comprehensively capturing the client experience of care across diverse health care settings. However, the emergence of similar domains across various health areas suggests there is the potential for developing a health area-agnostic approach to measuring client experience of care. The Larson framework proved useful in analyzing the measures examined in this review, offering a structured approach to measure appraisal. However, the broad categories within the framework do not completely account for the numerous features that hold significance for individuals during their health care journeys, as evidenced by the measures’ many quality domains that could not be easily categorized into the framework's domains but may still be relevant to the construct of client experience of care. To facilitate the development of a generalizable measure, it is crucial to further explore and define the construct of client experience of care and elucidate the constituent domains and sub-domains that are most important to people seeking care and actionable for health systems actors.

In the development of a novel measure for client experience of care, it is imperative to leverage routine health information systems (RHIS) and mHealth service modalities to comprehensively capture the entire care seeking journey. Routine health information systems have become essential tools for health systems strengthening in LMICs. However, using RHIS data for decision-making remains a challenge in many countries, in part, because of fragmented data collection tools and definitions.^107^ The introduction of a novel measure for client experience of care could enhance data-driven decision-making by bolstering the quality of information gathered through RHIS. Additionally, the popularity and use of digital and mobile health technologies continues to increase in LMICs, many of which involve direct client interaction, making it possible to now widely deploy a generalized measure of client experience of care.^108^ The ability to measure clients’ experiences and preferences across both time and a variety of health service modalities can provide a more nuanced understanding of the client experience from start to finish.

While the goal might be a single health area-agnostic measure of client experience of care, it is important to not let this ambition obscure the important differences in the delivery of different health services. Finding a balance between measuring a universal set of domains important for client experience and ensuring that those domains are relevant to the cultural and real-world needs of the particular context in which they are being deployed should remain the priority.

### Limitations

It is important to acknowledge certain limitations of this review. The studies included in this review were limited to those published within a specified time frame and retrieved from databases accessible to the authors, which may have introduced potential selection bias. Additionally, the heterogeneity of the identified studies in regard to their design and settings may limit the generalizability of these findings. We did not conduct a full systematic review, and the pragmatic phased nature of the review may mean that some relevant studies were excluded. The phased approach, however, allowed us to refine our understanding of the evidence map—and gaps—for a topic whose definition and scope resisted clear delineation at the outset.

## CONCLUSION

Patient-centered measures of health service quality have been developed and used in specific health areas and contexts, yet a comprehensive, cross-cutting measurement approach for client experience of care is needed if we are to advance our understanding of health service quality from the client’s perspective, conduct meaningful comparisons across different health care settings, and equip health systems with the data needed to drive person-centered improvements. The development of such a measure requires further conceptual refinement, including the constituent domains and sub-domains, and eventual pilot testing of a measurement tool. We propose a collaborative process in which key actors in the global health community, alongside the voices of clients in health systems, are heard and fed into usable, actionable, and valid measures of client experience of care. We see a more robust and cohesive approach to conceptualizing and measuring client experience as a necessary precursor to advancing toward person-centered health systems. We hope that advancing measurement approaches will unlock opportunities for a range of actors, from donors to national governments to community-based organizations, to integrate client experience measurement into their work, supporting more person-centered and responsive health systems.

## Notes

**Peer Reviewed**
